# The Application of a Case-Based Social Media–Assisted Teaching Method in Cariology Education：Comparative Study

**DOI:** 10.2196/29372

**Published:** 2021-08-13

**Authors:** Li Li, Xiaobin Liu, Zeyuan Chen, Liyuan Wang, Xiaoli Lian, Huiru Zou

**Affiliations:** 1 Tianjin Key Laboratory of Oral and Maxillofacial Function Reconstruction Tianjin Stomatological Hospital The Affiliated Stomatological Hospital of Nankai University Tianjin China; 2 No 2 Teaching & Research Department of Conservative Dentistry, Endodontics and Oral Medicine Tianjin Medical College Tianjin China; 3 School of Medicine Nankai University Tianjin China

**Keywords:** social media, case-based learning, cariology, dental cavity preparation, college students

## Abstract

**Background:**

Current cariology education based on the traditional teaching method faces a lot of challenges. Meanwhile, the COVID-19 pandemic caused an unprecedented disruption in medical education and health care systems worldwide. Innovation in the teaching mode of cariology education is required to change the situation.

**Objective:**

The goal of the research was to evaluate the application effects of a case-based social media–assisted teaching method in cariology education.

**Methods:**

Dental students of class 2019 were enrolled into the experimental group, while students of class 2018 served as control. A case-based social media–assisted teaching method was used in the experimental group, which included preclass activity via social media, additional discussion and practice process record in class, and questions and answers on the platform after class. The traditional teaching method, which consisted of conventional preparation before class, traditional lectures and demonstrations followed by students practice in class, and questions and answers step after class, was used in the control group. The teaching materials were the same in both groups. At the end of the program, students from both groups took cavity preparation skill evaluation tests. Questionnaires were tested on the case-based social media–assisted teaching group students anonymously. All data were analyzed using SPSS statistical software (version 22.0, IBM Corp).

**Results:**

The mean student cavity preparation skill evaluation scores was 82.51 (SD 6.82) in the experimental group and 77.19 (SD 5.98) in the control group (*P*<.05). The questionnaire response rate was 100%. Of those, 94.3% (100/106) of the students recommended the case-based social media–assisted teaching method in cariology education. The majority of the participants agreed that it helped them memorize the theoretical knowledge of cariology, facilitated in-depth discussion, improved their enthusiasm and initiative in learning, and enhanced the relationship between teachers and students (104/106, 98.1%). They also recognized that the classroom atmosphere was active (94/106, 88.7%).

**Conclusions:**

The case-based social media–assisted teaching method was beneficial in terms of learning, as demonstrated by the statistically significant improvement of the cavity preparation skill evaluation scores and satisfaction from attending students. This method could be used to supplement the teaching of cariology.

## Introduction

As one of the key subjects of clinical dentistry, cariology education plays an important role in dental education [1]. Students need to master not only theoretical knowledge but also operational skills in order to be able to resolve clinical problems encountered independently. Cariology education is still mainly based on the traditional teaching method, which faces challenges such as the demand of implementing didactic instruction into clinical training and the high occurrence of depression, anxiety, and stress encountered by students; the relationship between students and teachers need to be more interactive [2-5]. Meanwhile, the COVID-19 pandemic has caused an unprecedented disruption in medical education and health care systems worldwide [6]. Cariology education has also been greatly affected. An innovative teaching mode is required to improve teaching quality and cope with the impact of COVID-19 on teaching activities.

Case-based learning (CBL) is an effective teaching tool used in a variety of medical fields that links theory to practice by illustrating teaching points with actual clinical cases, promoting active and self-directed learning, clinical reasoning, and problem solving [7,8]. CBL has been encouraged by a number of universities and colleges. Most instructors are aware of the benefits of CBL, and as a result, the contents of courses are being reviewed and improved. Students appreciate that what is expected of them has been made clearer [9,10]. CBL has been used in Tianjin Medical College in cariology education for many years.

Nowadays, students use YouTube, Twitter, Instagram, and Facebook to access learning sources. Social media–assisted teaching in medical education appears to have increased and evolved during COVID-19 [11-14]. Information provided by these platforms is of varying quality, and it is hard for students to identify which sources are reliable. They need guidance from teachers, tutors, and supervisors. WeChat is a free app launched by Tencent in 2011 that has become one of the most popular communication tools in China, with more than 1 billion active users and coverage in more than 200 countries and regions worldwide. It provides instant messaging services; supports toll-free calls; allows sharing of audio files, pictures, and videos; and delivers other communication services. As new media, WeChat has an important impact on the daily life, learning, and interpersonal communications of students [15]. Previously, research on social media–assisted teaching focused on Twitter, Facebook, Second Life, and other platforms. Only a few studies recognized the educational function of WeChat [16,17]; teachers more often criticized students for spending time on smartphones and getting distracted. Using social media productively and achieving satisfactory results remains a big challenge [18].

Therefore, this study aims to explore a case-based social media–assisted teaching method based on real clinical caries cases via WeChat for dental undergraduate cariology education and demonstrate its feasibility and acceptability.

## Methods

### Participant Criteria and Ethical Approval

**Figure 1:**
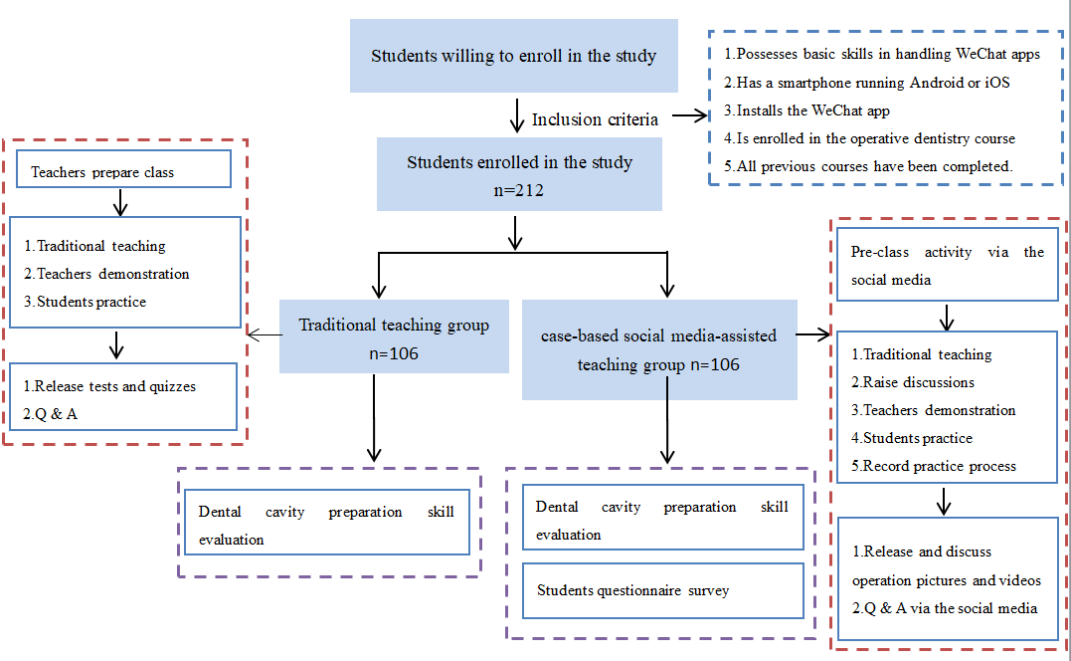
Flowchart of the process.

Dental students in the 2018 and 2019 classes majoring in stomatology joined in this study. Inclusion criteria were (1) possess basic skills in using WeChat apps, (2) have a smartphone running either Android or iOS, (3) have the WeChat app installed, (4) be enrolled in the cariology course, and (5) have completed all previous courses. Exclusion criteria were on long-term leave or having dropped out for any reason. Figure 1 shows a flowchart of the process. For standardization, the teachers were all from the No. 2 Teaching and Research Department of Conservative Dentistry, Endodontics, and Oral Medicine, of the Affiliated Stomatological Hospital of Nankai University with standard training and calibration. Ethical approval for this study was obtained from the Medical Ethics Committee of Tianjin Medical College (ID E20180008). Informed consent was obtained from all students before the program started.

### Experimental Design

Students of class 2018 were taught using the traditional teaching method and served as the control group, while students of class 2019 were taught using the case-based social media–assisted teaching method and served as the experimental group. Data were collected during the first semester of the 2018-2019 and 2019-2020 academic years. Students were divided into several small WeChat groups. More than 20 cases and problems on different topics were prepared by the faculty in the department. The teacher presented a brief explanation, followed by an introduction of a clinical case scenario accompanied by questions addressing the objectives of that part of the lecture. The students were asked to analysis the cases and make treatment plans accordingly. The experimental group used social media (mainly WeChat) within the whole training program. First, the teachers set up the experimental group before class using smartphones and invited students to join the group by releasing the group QR code. In addition to the same steps as the conventional preclass preparation of the traditional teaching group, the teachers would post plenty of pictures and short videos combined with text descriptions on the platform. Meanwhile, the teaching PowerPoint slides and preclass thinking questions would be released to the students. Second, the teachers reviewed the theoretical knowledge with students in class and led discussions about the preclass pictures and videos. The students then practiced according to the teachers’ instructions. An assistant teacher recorded and took pictures of the students’ practical positions, typical errors, and the cavity models prepared by the students. Finally, the teacher made comments and summarized after students’ practice. Third, the pictures and videos recorded by the assistant teacher would be released in the experimental group after class to inspire the students to discuss and work out problems, and the teachers would make comments. Excellent practice pictures and videos would be highlighted and shared with students. Students could learn independently, and peer evaluation was encouraged in order to stimulate students’ enthusiasm and participation. At the same time, the questions raised by students at any time would be answered through the platform by teachers.

The control group students experienced teaching activities according to the traditional teaching model. The same cases and problems of different topics were prepared by the faculty in the department and presented to students. Details were as follows: First, the teachers prepared according to the textbooks and syllabus before class and made teaching PowerPoint slides. Second, the teachers reviewed the theoretical knowledge in class first followed by video and live demonstrations. The students then practiced according to the teachers’ instructions, and the teachers made comments and summarized after the students’ practice. Third, teachers released tests and quizzes after class that required students to finish within certain times and answered the questions raised by students.

### Cavity Preparation Skill Evaluation

All students were given the same introductory lecture via PowerPoint presentation and the same demonstration from one trained teacher on the design and instrumentation of conventional class I cavity preparation. After that, students were asked to prepare class I cavities on the standard laser scanning model lower left first molar teeth (UNISN Inc). One tooth was prepared by each student within the 40 minute class. The teachers would then evaluate their cavity preparation skill levels using the Cavity Preparation Skill Evaluation System (CPSES). Briefly, the CPSES system scanned, evaluated, and scored the outline form and depth of prepared cavities against a theoretically ideal tooth model mounted on a special jig. The laser beam image was taken with 2 specialized digital cameras and processed with an image processing system. The converted image was transferred onto computer, and the contour and depth of the cavity was calculated. Finally both the 3D images of the prepared tooth and ideal tooth were displayed on the screen with the score and evaluation details, which could be printed out.

### Student Evaluation Survey

Meanwhile, in order to qualitatively analyze the impact of the case-based social media–assisted method on cariology education, student evaluation surveys were used as one of the main assessments. Interviews and group discussions were also employed in this study in order to get firsthand feedback and true voices from students. Examples of the questions were as follows: Are you satisfied with the case-based social media–assisted teaching method in cariology education? Are you interested in learning cariology? Did the case-based social media–assisted teaching method help you memorize caries-related knowledge and integrate the theoretical knowledge with clinical practice? The participants were asked to answer the questions anonymously at the end of the term.

### Statistical Analysis

All data were collected and analyzed with SPSS statistical software (version 22.0, IBM Corp). The Levene test was used to assess variance homogeneity for the paired *t* test. The Kolmogorov-Smirnov test was used to evaluate the normality of the sample distribution. The level of statistical significance was set at α=.05. The results of practical training were expressed as mean and standard deviation, and the data of the 2 groups were analyzed by paired *t* test. *P*<.05 indicated that the difference was statistically significant.

## Results

### Participant Analysis

There were 106 students each in class 2018 taught using the traditional teaching method, serving as the control group and class 2019 taught with the case-based social media–assisted teaching method, serving as the experimental group. The mean age of the students was 19.76 (SD 2.74) years in the experimental group versus 20.00 (SD 3.98) years in the control group (*P*=.97). A total of 78.3% (83/106) in the experimental group were women compared to 70.8% (75/106) in the control group (*P*=.39). No significant difference was seen between the 2 groups in terms of students’ previous scores (*P*=.69).

### Teacher-Student Interaction Via WeChat

**Figure 2:**
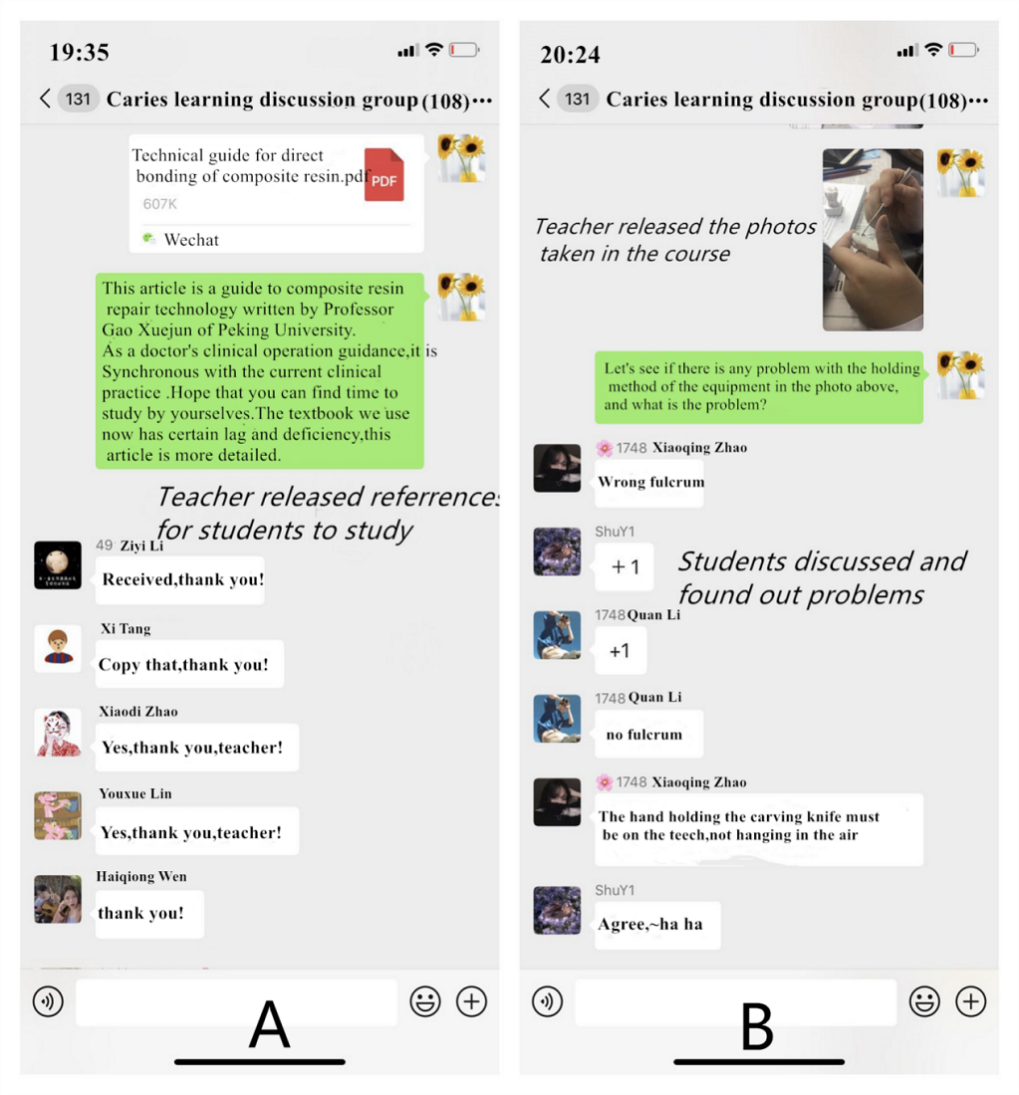
Teacher-student interaction on the social media platform: (A) teacher-released references for students’ self study and (B) teacher-released pictures and videos taken in the course with student discussion and resolution of problems.

Figure 2 shows the teacher-student interactions based on the WeChat social media platform. Pictures and short videos combined with text descriptions of clinical cases were presented and discussed on the platform. Over the period of the first semester of the 2019-2020 academic year, hundreds of chat-chat discussions took place on the WeChat social media platform. Notifications such as course notices, preclass questions, after-class study materials, and questionnaires were posted on this platform. Students felt that whenever they posted any question, there would be answers from the teachers or their classmates immediately. The WeChat learning tool was portable and instantly accessible.

### Cavity Preparation Skill Evaluation Results

**Figure 3:**
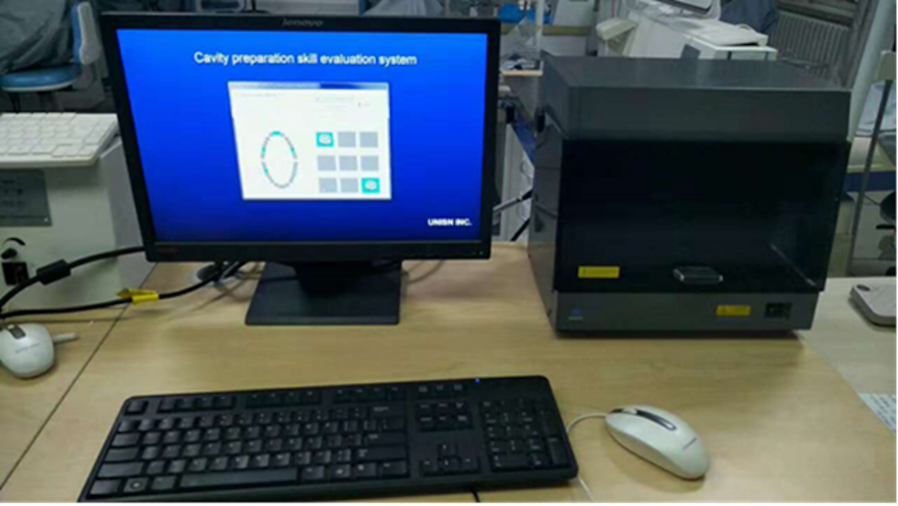
Cavity preparation skill evaluation system (CPSES).

**Figure 4:**
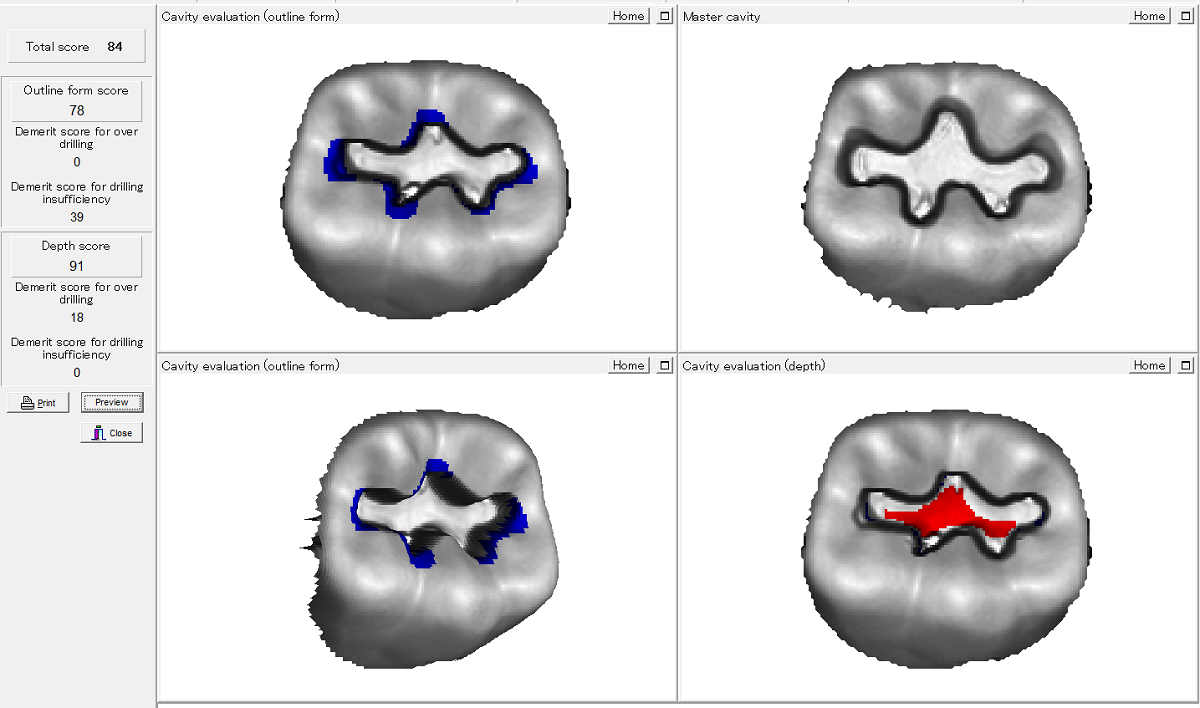
Assessment result revealed by cavity preparation skill evaluation system.

At the end of the training course, the dental cavities prepared by the students of both groups were assessed by CPSES (Figure 3). The results show that the scores of the experimental group were higher than those of the traditional teaching group. The scores of the experimental group were 82.51 (SD 6.82) and those of the control group were 77.19 (SD 5.98). There was a statistically significant difference between these groups (*P*<.05). Figure 4 shows the assessment results of the outline form and depth revealed by CPSES. There were significant improvements in the cavity outline form, retention form, smoothness, cavity depth, and cavity margin angulations of the experimental group-prepared dental cavities compared with the control group.

### Student Evaluation Survey Results

Completed questionnaires were returned from all 106 students of the experimental group. Thus, the response rate was 100%. A total of 94.3% (100/106) of the students were interested in the teaching method. The case-based social media–assisted teaching method improved students’ interest in learning, which helped them clearly understand each knowledge point, understand and memorize related knowledge, and master the key points of cavity preparation skills. It improved students’ enthusiasm and initiative in learning, and enhanced the relationship between teachers and students, as shown in Table 1. The majority of the interview and group discussion results were positive about the use of the case-based social media–assisted teaching method in cariology courses, which also confirmed the questionnaire results.

**Table 1:** Evaluation of teaching effect of experimental group.

Question	Yes		No	
	n	Constituent ratio (%)	n	Constituent ratio (%)
Are you satisfied with the case-based social media–assisted teaching method in cariology and restorative dentistry education?	100	94.3	6	5.7
Did the WeChat-assisted teaching method help you memorize related knowledge and integrate the theoretical knowledge with clinical practice?	104	98.1	2	1.9
Are you interested in learning cariology and restorative dentistry?	90	84.9	16	15.1
Was the classroom atmosphere active?	94	88.7	12	11.3
Are you satisfied with the degree of mastering cariology and restorative dentistry?	98	92.5	8	7.5
Did the teacher explain the material clearly?	96	90.6	10	9.4
Did the case-based social media–assisted teaching method help you understand the various knowledge points and memorize related knowledge?	106	100	0	0
Did the case-based social media–assisted teaching method improve learning enthusiasm and initiative?	104	98.1	2	1.9
Did the case-based social media–assisted teaching method enhance the relationship between teachers and students?	104	98.1	2	1.9

## Discussion

### Cariology Education Requirements

Dental caries are one of the most prevalent chronic diseases in the world. They have historically been considered the most important global oral health burden [19]. They are still a major health problem in many countries. Because of their high incidence rate, low treatment rate, high retreatment rate, and close relationship with general health, special attention still needs to be given in terms of prevention and treatment. Thus, the demand for delivering a quality cariology education for dental students is increasing.

Even though cariology is being taught in different ways in different countries, it is generally agreed that students should on graduation have a sound theoretical knowledge and understanding of cariology together with an adequate clinical experience to be able to resolve clinical problems encountered independently and without assistance. A professional attitude and behavior is also required. Students need to learn how to establish a trusting patient-dentist relationship and how to work with other members of the dental team [20,21]. Therefore students should continuously develop those skills including a thorough understanding and application of their moral, ethical, and legal responsibilities. CBL provides students patient details such as clinical signs and symptoms, a history of present illness, past history, and laboratory investigations. Students are actively involved in the discussion, interact with each other, and work together through the social media platform. CBL allows teachers more input into the direction of learning and induces learning on a deeper level. It allows students to be exposed to real-life case scenarios and enhances their analytical and thinking processes by this student-centered learning approach [22]. Cariology education is changing and adapting quickly to new technologies, and this has an impact directly on practical results.

### CBL Enhanced Theoretical-Clinical Integration and Communication

In this study, a case-based social media–assisted teaching method was applied to the cariology course. The effects were compared with a previous traditional teaching class. Among the students from the experimental group, 98.1% recognized that the case-based social media–assisted teaching method helped them memorize caries-related knowledge and integrate the theoretical knowledge with clinical practice. CBL improved the ability of the students to solve clinical problems, focusing on the integration of basic knowledge to clinical practice in the context of case-based learning [23]. At the same time, communication on the social platform between teachers and students encouraged students and increased their participation opportunities, providing personalized feedback according to the students’ performances. Previous research had stressed that knowing how much a student was involved in creating a learning environment would help teachers understand the student’s preference [24]. In another study, students were divided into several WeChat groups with 3 to 4 students per group. The teacher collected clinical data of patients in electronic records, images, and videos and delivered the materials to the WeChat groups before class with several questions. The students were asked to study the material individually and discuss the questions with each other in their small groups [25]. In this way, students could communicate and exchange ideas with their peers to solve the problems. Teachers could also share their ideas and experiences on the platform. WeChat became a communication tool for both teachers and students and also a platform for group work. Through this, core skills of evidence-based dental practice within the curriculum were highlighted. Students were able to identify uncertainty or gaps in understanding and formulating answerable questions, search for evidence using appropriate resources, and search for and use the most appropriate clinical guidelines. Teachers were able to deliver lifelong learning skills to the students.

### CBL Promoted Dental Cavity Preparation Skills

Dental cavity preparation and restoration technology involved in cariology are fundamental and closely related to clinical work. The traditional teaching mode of a practical training course mostly adopts the teaching mode of “teacher’s demonstration, student’s practice, teacher’s comments.” Due to the narrow space in the oral cavity, it is difficult for every student to observe the teacher’s demonstrations on the phantom head clearly. Additionally, students cannot effectively recognize the problems in their own operation. At the same time, due to the limitation of teaching time, teachers’ comments are often short, general, and cannot cover all aspects. The end of the training course means the end of the learning of practical skills. It is difficult for students to understand the biological, medical, basic, and applied clinical sciences in such a short period and apply knowledge to recognize caries and other dental hard tissue disorders and make decisions about their prevention and management. The social media–assisted teaching method is very helpful in carrying out teaching demonstrations. Before class, the teacher records the key operation steps and precautions on small videos or takes pictures for students to preview. This resolves time and space limitations of traditional teaching and learning methods, cultivates students’ independent learning ability, and meets the personalized learning needs of different levels of students. The theoretical knowledge test scores of social media–assisted teaching groups were significantly higher than those of traditional teaching groups. Previous studies also showed that social media had a generally positive impact on teaching effect, consistent with the results of this study [17,18,26]. However, most of those studies only used theoretical tests, questionnaires, students’ feedback to evaluate the teaching effects, lacking objective evaluation indicators. In recent years, we have adopted CPSES to make assessments through the computer 3D scanning device and image processing system. The results are objective and fair, excluding the interference of subjective factors, making the assessments more scientific [27,28]. This study continued to use this evaluation system to objectively evaluate the effect of social media–assisted teaching in the dental cavity preparation skill training. The results showed that students’ cavity preparation scores in the social media–assisted teaching group were significantly higher than those in the traditional teaching group.

### CBL Extended Traditional Teaching

Social media–assisted teaching was a powerful supplement to the traditional classroom teaching, which significantly enhanced the teaching effect. After class, the typical classroom operation videos and pictures taken by the assistant teacher were sent to the students immediately. Students had a clearer understanding of their own problems and learned through the strengths and weaknesses of others. Yang et al [29] conducted a study on teaching fundamental nursing skills with smartphone-based video feedback to facilitate teaching. Results showed that video feedback was an effective way to improve nursing students’ academic performance and professional skills. In our study, in the process of gypsum cavity preparation and phantom head cavity preparation, the operation fulcrum was emphasized. Students were required to have a stable fulcrum when they operated. Beginners often easily ignored this problem. In the social media–assisted teaching group, teachers sent pictures of students practicing without a fulcrum or with a wrong fulcrum to the students after class. This action formed a strong visual stimulation for students and enhanced their memory about this point. Teachers asked questions and encouraged students to discuss and collaborate with others. Some studies have confirmed that online teaching methods could enable students to get timely feedback from teachers and other students in the learning process, and students were familiar with online communication methods and participated in discussions more actively [13,29]. Teaching social and communicative competencies has become an important part of undergraduate dental education [20]. Studies have also shown that social media promoted the formation of a student-centered teaching mode by providing information and interactive feedback anytime and anywhere, which significantly improves the proportion of students actively participating in learning and enhanced the satisfaction of students [13]. These results were also confirmed in our study. As the students had rarely been exposed to an activity like CBL, it was observed that some students participated actively while some remained passive in the traditional method [30]. In this study, the WeChat platform made the learning mode more diversified, enabled students to grasp the key points of practical training more solidly, and effectively extended the traditional teaching. The platform also reduced the anxiety and tension of face-to-face communication between students and teachers, and made communication occur more smoothly. WeChat was helpful in eliminating the possible barriers between teachers and students and effectively enhanced the relationships between teachers and students. The results of our study showed that students were actively participating in the whole teaching and learning process, communicating and interacting with peers as well as faculty members on the social platform. Meanwhile, extra financial costs of maintaining and updating modules were not required, which made it more popular and likely to be applied in other fields.

Case-based social media–assisted teaching in cariology education promoted students’ learning and mastering of advanced treatment technology and the expansion of cutting-edge knowledge in clinical treatment. Teachers used social media to provide multimedia teaching materials, such as pictures, audios, and videos, so that students could learn in an all-round way more intuitively and vividly through the network anytime and anywhere, providing a convenient way for teaching [31]. In order to better adapt to the needs of clinical technology development, cariology teaching should also constantly update the knowledge system so that students can use the latest medical equipment, materials, and methods. Teachers can use social media to demonstrate advanced treatment technology to students (eg, minimally invasive comprehensive prevention and treatment of caries, preparation and filling technology of cavities under the microscope) so that students’ learning is not out of line with clinical practice, which makes up for the slight lag of current textbooks. Most of the students in medical colleges have not received systematic scientific research training. They lack the ability to access knowledge effectively through the internet. Teachers could push new authorized theoretical knowledge directly to students on social media so that students can learn new knowledge more effectively. Through the study of current advanced knowledge and technology, students can realize the difference between practical training operation and current clinical operation. They can also discuss the difference between practical training operation technology and clinical advanced technology, which will stimulate students’ learning initiative and enthusiasm, improving the learning effects. The improvement of the learning effect would certainly improve the competitiveness of students. Several studies have confirmed that the use of social media promoted development of effective work relationships and were helpful for a successful professional career [32,33]. Inspired by the clinical cases delivered by teachers through social media, students gained knowledge of the impact of restorative procedures on mucosa, periodontal tissues, occlusion, and oral function. They also learned the skills of selecting and handling appropriate restorative materials considering physical and chemical properties, biocompatibility, and longevity and selecting and carrying out operative techniques appropriate for both material and case. Through clinical cases introduced on social media, students achieved skills of deciding when, how, and to what extent to remove carious tissue before the placement of a restoration, considering the restorability of the tooth, preservation of tooth structure, and pulp vitality. Meanwhile, they were also aware of the health economic aspects of restorative therapy and patient privacy protection issues. It was found that social media–assisted teaching improved students’ independent learning, learning interest, enthusiasm, and ability to cooperate with peers, thus promoting education reform [34]. Therefore, students would have a perceptual understanding of the training courses to be learned.

### Limitations

First, the study population was restricted to one medical college in China. Therefore, it might limit the generalizability of the study. Further studies carried out in other colleges or universities would be expected, which would provide stronger evidence on the effectiveness of the case-based social media–assisted teaching method.

Another limitations of the study was the lack of randomized controlled design. The premises were that education in experimental design should be undertaken with an awareness of educational principles. Unlike when using laboratory animals, more restricted ethical considerations needed to be considered. Students deserved to learn with the most suitable and most effective teaching method. Therefore, we decided to enroll the entire 2019 class of students into the experimental group and chose the previous class with the traditional teaching method as control. Since students knew very little about cariology and had no capability to prepare dental cavities before class, the pretest posttest quasi-experiments were not carried out in this study. Hence, the results of this study should be interpreted with caution. We hope that the case-based social media–assisted teaching method will be used to help students be equipped to provide appropriate care for the most widespread oral disease.

### Conclusion

The case-based social media–assisted teaching method was beneficial in cariology education in terms of learning, as demonstrated by the statistically significant improvement of CPSES and satisfaction of the attending students. The application of the case-based social media–assisted teaching method in cariology education helped students uptake the theoretical knowledge, ignited their enthusiasm and motivation in learning, and relieved their stress. The pilot study revealed that the case-based social media–assisted teaching method could be used to supplement the teaching of cariology and is worth further study and application.

## Abbreviations

CBL: case-based learning
CPSES: Cavity Preparation Skill Evaluation System

## References

[1] Hilton TJ (2013). Summitt’s Fundamentals of Operative Dentistry: A Contemporary Approach.

[2] Tikhonova S, Girard F, Fontana M (2018). Cariology education in Canadian dental schools: Where are we? Where do we need to go?. J Dent Educ.

[3] Raphael SL, Foster Page LA, Hopcraft MS, Dennison PJ, Widmer RP, Evans RW (2018). A survey of cariology teaching in Australia and New Zealand. BMC Med Educ.

[4] Basudan S, Binanzan N, Alhassan A (2017). Depression, anxiety and stress in dental students. Int J Med Educ.

[5] Oderinu OH, Adegbulugbe IC, Orenuga OO, Butali A (2020). Comparison of students' perception of problem-based learning and traditional teaching method in a Nigerian dental school. Eur J Dent Educ.

[6] Tolsgaard MG, Cleland J, Wilkinson T, Ellaway RH (2020). How we make choices and sacrifices in medical education during the COVID-19 pandemic. Medical Teacher.

[7] Kaur G, Rehncy J, Kahal KS, Singh J, Sharma V, Matreja PS, Grewal H (2020). Case-based learning as an effective tool in teaching pharmacology to undergraduate medical students in a large group setting. J Med Educ Curric Dev.

[8] Alhazmi A, Quadri MFA (2020). Comparing case-based and lecture-based learning strategies for orthodontic case diagnosis: a randomized controlled trial. J Dent Educ.

[9] Alsunni AA, Rafique N (2021). Effectiveness of case-based teaching of cardiovascular physiology in clinical pharmacy students. J Taibah Univ Med Sci.

[10] Jacob SA, Dhing OH, Malone D (2019). Perceptions of Australian and Malaysian educators in an undergraduate pharmacy program on case-based learning. Am J Pharm Educ.

[11] Wanner GK, Phillips AW, Papanagnou D (2019). Assessing the use of social media in physician assistant education. Int J Med Educ.

[12] Maggio LA, Leroux TC, Artino AR (2019). To tweet or not to tweet, that is the question: a randomized trial of Twitter effects in medical education. PLoS One.

[13] Javaeed A, Kibria Z, Khan Z, Ghauri SK (2020). Impact of social media integration in teaching methods on exam outcomes. Adv Med Educ Pract.

[14] Laurentino Lima D, Nogueira Cordeiro Laurentino Lima R, Benevenuto D, Soares Raymundo T, Shadduck PP, Melo Bianchi J, Malcher F (2020). Survey of social media use for surgical education during COVID-19. JSLS.

[15] Wang J, Gao F, Li J, Zhang J, Li S, Xu G, Xu L, Chen J, Lu L (2017). The usability of WeChat as a mobile and interactive medium in student-centered medical teaching. Biochem Mol Biol Educ.

[16] Luan H, Wang M, Sokol RL, Wu S, Victor BG, Perron BE (2020). A scoping review of WeChat to facilitate professional healthcare education in Mainland China. Med Educ Online.

[17] Zhang W, Li Z, Li Z (2019). WeChat as a platform for problem-based learning in a dental practical clerkship: feasibility study. J Med Internet Res.

[18] Li B, Wu Y, Jiang S, Zhai H (2018). WeChat addiction suppresses the impact of stressful life events on life satisfaction. Cyberpsychol Behav Soc Netw.

[19] GBD 2017 Disease and Injury Incidence and Prevalence Collaborators (2018). Global, regional, and national incidence, prevalence, and years lived with disability for 354 diseases and injuries for 195 countries and territories, 1990-2017: a systematic analysis for the Global Burden of Disease Study 2017. Lancet.

[20] Meyer-Lueckel H, Opdam NJM, Breschi L, Buchalla W, Ceballos L, Doméjean S, Federlin M, Field J, Gurgan S, Hayashi M, Laegreid T, Loomans BAC, Lussi A, Lynch CD, Pallesen U, Peumans M, Toth Z, Wilson NHF (2019). EFCD Curriculum for undergraduate students in Integrated Conservative Oral Healthcare (ConsCare). Clin Oral Investig.

[21] Lichtenstein NV, Haak R, Ensmann I, Hallal H, Huttenlau J, Krämer K, Krause F, Matthes J, Stosch C (2018). Does teaching social and communicative competences influence dental students' attitudes towards learning communication skills? A comparison between two dental schools in Germany. GMS J Med Educ.

[22] Kaur G, Rehncy J, Kahal KS, Singh J, Sharma V, Matreja PS, Grewal H (2020). Case-based learning as an effective tool in teaching pharmacology to undergraduate medical students in a large group setting. J Med Educ Curric Dev.

[23] Bi M, Zhao Z, Yang J, Wang Y (2019). Comparison of case-based learning and traditional method in teaching postgraduate students of medical oncology. Medical Teacher.

[24] Pyle E, Hung W (2019). The role of subject presence type on student motivation in a PBL learning environment. Adv Health Sci Educ Theory Pract.

[25] Zeng F, Deng G, Wang Z, Liu L (2016). WeChat: a new clinical teaching tool for problem-based learning. Int J Med Educ.

[26] Tu S, Yan X, Jie K, Ying M, Huang C (2018). WeChat: an applicable and flexible social app software for mobile teaching. Biochem Mol Biol Educ.

[27] Zou H, Jin S, Sun J, Dai Y (2016). A cavity preparation evaluation system in the skill assessment of dental students. J Dent Educ.

[28] Zou H, Jin S, Sun J, Dai Y (2016). Cavity preparation skill evaluation system for assessing junior dental students. Med Educ.

[29] Yang X, Xie R, Chen S, Yu W, Liao Y, Krewski D, Wen SW (2019). Using video feedback through smartphone instant messaging in fundamental nursing skills teaching: observational study. JMIR Mhealth Uhealth.

[30] Gupta K, Arora S, Kaushal S (2014). Modified case based learning: our experience with a new module for pharmacology undergraduate teaching. Int J App Basic Med Res.

[31] Montag C, Becker B, Gan C (2018). The multipurpose application WeChat: a review on recent research. Front Psychol.

[32] Vande VL, Ryder H, Best J (2021). Maximizing career advancement during the COVID-19 pandemic: recommendations for postgraduate training programs. Acad Med.

[33] Rajeh MT, Sembawa SN, Nassar AA, Al Hebshi SA, Aboalshamat KT, Badri MK (2021). Social media as a learning tool: dental students' perspectives. J Dent Educ.

[34] Guraya SY, Al-Qahtani MF, Bilal B, Guraya SS, Almaramhy H (2019). Comparing the extent and pattern of use of social networking sites by medical and non medical university students: a multi-center study. Psychol Res Behav Manag.

